# Paying for artificial intelligence in medicine

**DOI:** 10.1038/s41746-022-00609-6

**Published:** 2022-05-20

**Authors:** Ravi B. Parikh, Lorens A. Helmchen

**Affiliations:** 1 Perelman School of Medicine, University of Pennsylvania, Philadelphia, PA USA; 2 Leonard Davis Institute of Health Economics, University of Pennsylvania, Philadelphia, PA USA; 3 Milken Institute School of Public Health, The George Washington University, Washington, DC USA

**Keywords:** Health policy, Health care economics

## Abstract

Over the past 7 years, regulatory agencies have approved hundreds of artificial intelligence (AI) devices for clinical use. In late 2020, payers began reimbursing clinicians and health systems for each use of select image-based AI devices. The experience with traditional medical devices has shown that per-use reimbursement may result in the overuse use of AI. We review current models of paying for AI in medicine and describe five alternative and complementary reimbursement approaches, including incentivizing outcomes instead of volume, utilizing advance market commitments and time-limited reimbursements for new AI applications, and rewarding interoperability and bias mitigation. As AI rapidly integrates into routine healthcare, careful design of payment for AI is essential for improving patient outcomes while maximizing cost-effectiveness and equity.

Artificial intelligence (AI)—broadly defined as the use of computing power to evaluate many data points and recommend or take an action in real-time—promises faster, cheaper, and more accurate diagnosis and prognosis of disease beyond what is possible by human judgment alone. Between 2015 and 2020, the United States Food and Drug Administration (FDA) and the European Commission (EC) cleared 222 and 240 AI devices, respectively, for clinical use, often under the “software as a medical device” or similar designation^1^. In a key step towards broader dissemination of AI, in August 2020, the US Centers for Medicare & Medicaid Services (CMS) announced its intention to provide coverage for the first AI-specific Common Procedural Terminology (CPT) code and the creation of the first New Technology Add-On Payment (NTAP) for an AI device—both historical precedents for reimbursement of AI devices^2^. Currently, CMS reimburses the use of at least 8 AI devices (Table 1). As the experience with reimbursement of non-AI medical devices in the past has shown, reimbursement of AI devices could encourage or inhibit the wider use of AI in medicine. However, per-use AI reimbursement may result in overuse—an undesirable outcome of AI reimbursement policy. Given this concern, we describe five alternative and complementary reimbursement approaches for AI in healthcare.

**Table 1:** Selected AI devices that are reimbursed by US Medicare.

Manufacturer	Technology	Description	Payment mechanism	Year reimbursement granted
Digital diagnostics	IDX-DR	Deep learning algorithm to diagnose diabetic retinopathy from fundoscopic images in the outpatient setting	CPT	2020
viz.ai	Viz LVO	Radiological computer-assisted triage and notification software that analyzes CT images of the brain and notifies hospital staff when a suspected large-vessel occlusion (LVO) is identified	NTAP	2020
Rapid AI	Rapid LVO	AI-guided medical imaging acquisition system intended to assist medical professionals in the acquisition of cardiac ultrasound images.	NTAP	2020
Caption health	Caption guidance		NTAP	2021
viz.ai	Viz SDH	Radiological computer-assisted triage and notification software that analyzes CT images of the brain and notifies hospital staff when a suspected subdural hematoma is identified	NTAP	2022 (candidate)
Rapid AI	Rapid aspects	Computer-aided diagnostic device characterizing brain tissue abnormalities on brain CT images	NTAP	2022 (candidate)
AIDoc	Briefcase for PE	Radiological computer-assisted triage and notification software that analyzes CT images of the chest and notifies hospital staff when a suspected pulmonary embolism is identified	NTAP	2022 (candidate)
PROCEPT BioRobotics Corporation	The AQUABEAM system	Autonomous tissue removal robot for the treatment of lower urinary tract symptoms due to benign prostatic hyperplasia (BPH).	NTAP	2020

## AI in the current reimbursement landscape

AI applications can be grouped into classes: assistive, augmentative, and autonomous. While certain types of AI may warrant slight modifications to payment policy, our general principles are applicable to all AI-based technologies. AI-based technologies have the potential to confer clinical benefits to patients and to raise physician productivity by increasing the time available for other tasks related to direct patient care. Viz LVO, a computer-based system that automatically detects large-vessel cerebrovascular occlusions from CT scans using an AI method known as a convolutional neural network, has been associated with a 45% reduction in time to potentially life-saving intervention compared to routine practice by speeding reading time and alerting radiologists early to potential large-vessel stroke^3^. IDX-DR, another AI device based on fundoscopic images, identifies diabetic retinopathy—the leading cause of vision loss in adults globally—with sensitivity and specificity greater than 90%^4^. IDX-DR is usable in the primary care setting and thus may expand access to high-quality screening for diabetic retinopathy, which only 30–60% of Medicare beneficiaries with diabetes receive^5^. AI devices are valuable to patients by accelerating the detection of pathology or predicting future adverse events more accurately, thus facilitating timely interventions and informing patients’ healthcare choices, which in turn may improve outcomes. AI devices are valuable to clinicians by diagnosing or predicting pathology with potentially better accuracy and speed than achieved by clinician judgment alone, thus improving efficiency.

In 2021, the FDA released its Artificial Intelligence and Machine Learning Software as a Medical Device Action Plan, which proposed a vision of total product lifecycle-based regulatory oversight of AI devices^6^. This plan includes proposed standards for algorithmic bias mitigation, transparency, revisions and updates of algorithms, and real-world performance monitoring. The International Coalition of Medicines Regulatory Authority released similar guidance in 2021^7^. These documents also provide a pathway for AI software applications to become potentially patentable when claimed as a method or process with sufficient specificity.

Reimbursement policies are important means of disseminating the benefits of AI across the healthcare system. CMS can use the FDA’s regulatory standards to determine coverage of innovative technologies such as AI; however, no formal guidance regarding standards for CMS coverage of AI has been released to date. Broader proposals to ensure CMS coverage of designated breakthrough devices such as AI, including the Medicare Coverage for Innovative Technologies (MCIT) final rule, were later withdrawn^8^. Furthermore, CMS and other US payers have made significant strides in moving from the current fee-for-service payment model to value-based payment models, which reimburse providers and services based on outcomes. Indeed, CMS leadership has announced a goal that every Medicare beneficiary and a majority of Medicaid beneficiaries will be in a value-based care arrangement by 2030^9^. Current efforts within the traditional (fee-for-service based) Medicare program, including the Merit Incentive Payment System (MIPS) and the Medicare Shared Savings Program (MSSP), offer incentives for high-quality care; in fact, the MIPS includes AI-based diagnostics as meeting standards for certain quality metrics such as diabetic eye exams. These payment efforts are complemented by existing efforts not related to reimbursement to improve healthcare quality and value, such as the measures captured by the Healthcare Effectiveness Data and Information Set (HEDIS). The impending transition to value-based payment arrangements and the rapid development and deployment of medical AI leave little time for regulatory agencies and payers such as CMS to establish reimbursement guidance.

## Current state of AI reimbursement in healthcare

As an initial policy, CMS has adopted per-use payments for AI, primarily by covering AI-specific CPT codes created by the American Medical Association CPT Editorial Panel or by establishing NTAPs for AI devices—two common forms of reimbursement for medical devices. NTAP payments, which are part of Medicare’s Inpatient Prospective Payment System (IPPS), provide additional reimbursements to hospitals above the standard Medicare Severity Diagnosis-Related Group (MS-DRG) payment amount for new technologies that raise a provider’s cost of treating an illness episode above the existing episode-based payment and that provide a “substantial clinical benefit”^10^. The Viz LVO NTAP adds a maximum of $1,040 to a hospital’s payment for managing a stroke episode, which is meant to cover the cost of operating room use, nursing, supplies, as well as laboratory and imaging services. CMS has also established new CPT codes for each use of certain AI devices; such CPT codes are typically used when billing for AI services used in outpatient care but could be used to seek reimbursement for inpatient care as well. Reimbursement for CPT codes is often determined based on Relative Value Units (RVUs) assigned under the Medicare Physician Fee Schedule, which is composed of resource costs associated with physician work, practice expenses, and liability insurance. Such RVUs are adjusted based on geographic medical costs and wage differentials. There are limitations to the use of such codes: for example, retinal telescreening CPT codes may only be used for screening and not for monitoring diabetic retinopathy. In contrast, in Europe, current systems do not recognize AI software as a separately reimbursable expense, and AI solutions are routinely not covered by health insurance premiums^11^.

## Per-use AI reimbursement may lead to overuse

NTAP and coverage of CPT codes are common reimbursement models for traditional medical devices, although not all CPT codes will be covered by Medicare. Yet, per-use payment models fail to recognize the scalability and automation of AI that should inform payment policy. AI can be integrated rapidly into software and thus can impact the lives of many more patients at a much lower marginal cost than is the case for traditional medical devices. In addition, AI applications can generate diagnostic or prognostic output automatically without a clinician’s decision. As such, per-use payments run the risk of reimbursing AI at a much higher volume than traditional medical devices.

Most AI devices are meant to improve efficiency in diagnosis or workflow, and thus aim to complete tasks that were performed by clinicians before. For instance, traditionally, medical images were interpreted by clinicians, who must undergo years of specific training and must accrue many hours practicing the skill. In contrast, AI devices can be set up to serve as automatic, independent screeners for a pathology, upon which a radiologist or ophthalmologist would offer oversight. To be sure, clinicians may spend time to interpret an assistive AI application or explain AI results to a patient. However, in an ideal scenario, the efficiency gains of an AI application considerably decrease the number of clinician hours used for diagnosis, prognosis, and information synthesis. Indeed, the current use of image-based AI is layered upon existing procedures, such as CT screening and ophthalmologic examination, which is already covered through the procedure- or episode-based payments in the US and Europe such as MS-DRGs or alternative payment models, including bundled payments. This increased efficiency could increase the number of billable diagnostic procedures, resulting in higher overall spending, more frequent detection of disease, more treatment, and better patient health.

As CMS has recognized in its 2022 Physician Fee Schedule, AI may improve access to Medicare-covered services, such as diabetic retinopathy screening, in rural and/or underserved areas^12^. As such, reimbursement structures should not incentivize the underuse of evidence-based AI. However, the same Schedule acknowledges the possibility that AI poses a greater risk of overutilization than most devices and offers greater potential for fraud, waste, and abuse. Per-use reimbursement for AI may encourage overuse when the payment per-use exceeds the monetary value of the medical net benefit that the use confers to patients and of the productivity gain that the use confers to clinicians. While many clinicians may not engage in an explicit comparison of per-use reimbursement and the average per-use cost to inform treatment decisions, payers and administrators may track and communicate instances of repeated over- or underuse to clinicians and tie reimbursement to these indicators. As with most medical technologies, overuse of AI may lead to overdiagnosis and trigger healthcare spending of little medical value, with a value measured as the improvement in patient outcomes expressed in monetary units. Granted, factors other than payment rates (e.g., coverage determinations, utilization management) also have a role to play in overutilization. However, whether these levers are used depends on the underlying reimbursement structure, specifically per-use payment vs. alternative reimbursement methodology, chosen.

Reimbursement of AI is nascent, and it may take years to evaluate whether CMS’s initial per-use reimbursement policies have led to the overuse of medical AI. However, experience with other devices related to imaging and procedural services suggests that per-use reimbursement encourages overuse^13,14^. For example, reductions in fee-for-service reimbursement have been shown to be associated with decreases in unnecessary radiology utilization in chronic diseases such as stroke^15^.

Moreover, over time the cost per-use will decline with further advances in information technology, such as declines in cloud storage costs and increases in computational capacity. In a competitive environment with flexible price-setting, these cost savings would be passed on to payers. In the absence of this mechanism, and even more acutely than for traditional medical devices, the changes in the cost of medical AI must be met by periodic adjustments in per-use reimbursement rates. If not, payers risk overspending on AI.

Drawing on examples from traditional medical devices and pharmaceutical agents, we describe five alternative and complementary reimbursement approaches for AI in healthcare.

## Forgoing separate reimbursement of AI devices

One potential alternative reimbursement approach is to not offer separate reimbursement of AI at all. Arguably, AI should be deployed only if health systems achieve significant savings or generate significant additional revenue from AI even without separate payer reimbursement for AI, via reduced preventable utilization, faster discharge, or earlier or more frequent diagnosis of a disease that triggers the performance of additional subsequent procedures. Other innovative device manufacturer-health system contracts may obviate the need for payer reimbursement by allowing AI’s efficiency gains to translate into cost savings or revenue for both manufacturers and health systems, without the need for separate payer reimbursement. For example, device manufacturers could specify a volume-based price to health systems and offer discounts or rebates if clinical or economic outcomes are not met. One large manufacturer of cardiac resynchronization therapy has offered rebates of up to 45% if revisions are necessary after implantation. In other gain-sharing models, device manufacturers sell AI devices at a discount but share in the revenue generated from that device. Certain manufacturers have agreed to sell devices such as antimicrobial catheters for lower prices if a hospital agrees to share savings from the prevention of urinary tract infections in an outcomes-based contract. Given that AI offers similar cost-saving potential via the prevention of adverse events, gain-sharing models between AI manufacturers and health systems may be similarly applicable. A strength of this approach is that, compared to governments and payers, health systems, hospitals, and clinicians may be better judges of the value of AI, including gains in patient or provider satisfaction that may not result in savings, and respond more quickly to newer AI entrants into the market than payers might.

Nevertheless, forgoing reimbursement of AI is unlikely to be a viable strategy for all AI devices. Separate reimbursement of AI by payers has advantages. Most importantly, as the experience with telemedicine has shown, inadequate reimbursement for AI in its infancy may discourage the utilization of technology that improves patient outcomes and lowers costs in the intermediate to long term^16^. Additionally, AI devices may benefit patients in ways that are not reflected by a health system’s cost savings or rise in revenue. For example, AI-based systems may assist clinicians in reviewing patient records and synthesizing and highlighting clinically relevant data^17^. Such devices may reduce the time required for physician chart review by 20%. While these improvements may eventually result in cost savings or productivity gains for healthcare providers, they immediately confer a benefit to patients by improving wait time and other patient-centered metrics.

## Incentivize outcomes instead of volume

To encourage the development and deployment of effective AI applications, payers could reward health systems that achieve patient-centered or process-related outcomes that are responsive to AI. For example, payers could offer higher reimbursements to healthcare providers for stroke detection AI devices if post-marketing studies continue to demonstrate a positive effect on validated quality metrics in stroke care, such as specific door-to-puncture targets for interventional procedures or patient functional improvement.

European payers have already shown a willingness to experiment with value-based payments for other high-cost services^18^. One notable example is the UK’s.

Commissioning for Quality and Innovation (CQUIN) tariff system. The CQUIN system allows health commissioners to hold back 2·5% of the payment for hospital treatment contingent on pre-specified outcomes. These mechanisms are potentially ripe to apply to episodes of care using AI-based technologies. Other examples of outcomes-based purchasing come from the US CMS’s experience with high-cost pharmaceuticals. CMS’s Medicaid program, primarily intended for low-income beneficiaries, has implemented a Drug Rebate Program that requires a drug manufacturer rebate in exchange for a place on their formulary^19^. This rebate is greater for higher-value drugs, assessed by outcomes-based measures. This strategy could be used to align payment for AI devices with their value.

## Advance market commitments for new AI solutions

For AI technologies that offer considerable patient benefit and adhere to regulatory standards, payers and regulatory bodies could offer advance market commitments for AI that responds to specific healthcare delivery challenges. Akin to the “X Prize” model, this model offers a premarket monetary prize commensurate with the social value of overcoming the healthcare delivery challenge. This reimbursement mechanism has been used to foster the development of AI algorithms that forecast COVID-19 infection rates^20^. The prize would be awarded to the first entrant whose deliverable met the requirements set forth in the solicitation. In return, the developer would make the code used in the AI application publicly available immediately to encourage as wide a circle of competitors as possible to develop improved follow-on AI.

## Time-limited add-on reimbursements for novel AI

Another strategy to cover the cost of developing novel AI tools are time-limited add-on payments. For example, CMS’s transitional drug add-on payment (TDAP) is meant to cover the cost of new pharmaceuticals, such as drugs for end-stage renal failure, not yet accounted for in bundled or episode-based payments. The add-on payment facilitates beneficiary access to certain qualifying, new, injectable, or intravenous drugs and biologics, and allows payers such as CMS to gather sufficient data to incorporate the new therapy into the bundle and adjust the base payment rate. The therapy is included in the bundle at the end of the 2–3-year TDAP period. This strategy could be feasibly used to reimburse new AI entrants for a similar time-limited period. If the AI application had been incorporated into the standard of care, its cost could be rolled into episode-based payments afterward.

## Reward interoperability and bias mitigation

AI improves population health through early detection and screening; however, this benefit is maximally realized if AI can work across several health systems or health settings. In the context of AI in medicine, interoperability refers to the applicability of the AI results across multiple populations, even when training datasets were drawn from different geographic domains or populations with different demographic or disease characteristics. For instance, an AI tool to predict sepsis that was based on data drawn from a single institution may fail to perform as well at another clinical institution^21^. In general, AI-based technologies typically work best for the populations and medical conditions for which they were trained, which often reflect current patterns of healthcare coverage and use. AI-based technologies thus may fail to confer the same benefit for populations and medical conditions that were underrepresented in the training set – including racial and ethnic minorities and rare or underdiagnosed medical conditions^22^. It is for this reason that the FDA has prioritized developing methodologies to detect and mitigate bias in AI and using equitable performance as a key regulatory metric for AI^6^. To further this goal, payers could provide financial incentives for AI devices to be generalizable across health systems and patient populations that stand to benefit substantially from their use. For example, payers could provide greater reimbursements for AI devices that demonstrate interoperability and applicability to multiple settings and patient groups in premarket testing. The FDA’s recent Digital Health Innovation Action Plan, issued in 2017, launched a precertification program to streamline AI devices for premarket review; this precertification program could emphasize interoperability and coverage as a path toward CMS reimbursement. Alternatively, national payers could crowdsource proposals through innovation challenge competitions for AI-based tools, providing financial or regulatory incentives for AI technologies that meet pre-defined standards for interoperability and for bias mitigation.

## Conclusion

AI in medicine offers the potential to improve patient outcomes, provider productivity, and equity in healthcare delivery. The European Commission’s Executive Agency for Small and Medium-sized Enterprises (EASME), the FDA’s Digital Health Center for Excellence, and CMS all have recognized reimbursement for AI as a major priority. The approaches we have described above can guide reimbursement policy to prioritize value and disincentivize overuse. In combination with careful regulation, a reimbursement model that recognizes AI’s rapid scalability and automation will reward value rather than volume—sending an important signal as AI rapidly integrates into routine healthcare to improve patient outcomes.

### Reporting Summary

Further information on research design is available in the Nature Research Reporting Summary linked to this article.

## Supplementary information

Reporting Summary

## Data Availability

Data sharing is not applicable to this article as no datasets were generated or analysed during the current study.
